# A computed tomography-based radiomics-clinical model incorporating left atrial and proximal pulmonary vein features predicts recurrence after radiofrequency catheter ablation of atrial fibrillation: a multicenter study

**DOI:** 10.3389/fmed.2026.1935193

**Published:** 2026-09-16

**Authors:** Hui-Xian Jin, Hui Li, Shaoyu Han, Ruyi Wang, Huanqing Xu, Mingya Zhang, Zhen Chen

**Affiliations:** 1 Department of Cardiology, Taixing People’s Hospital Affiliated to Yangzhou University, Taixing, Jiangsu, China; 2 Department of Cardiology, School of Medicine, Yangzhou University, Yangzhou, Jiangsu, China; 3 Department of Cardiology, Xuzhou Medical University, Xuzhou, Jiangsu, China; 4 School of Medical Information Engineering, Anhui University of Chinese Medicine, Hefei, Anhui, China; 5 School of Medical Information Engineering, Wannan Medical University, Wuhu, Anhui, China

**Keywords:** atrial fibrillation, coronary computed tomography angiography, late recurrence, left atrial complex, radiofrequency catheter ablation, radiomics

## Abstract

**Objective:**

To develop and validate a CCTA-based radiomics-clinical model for predicting late recurrence after radiofrequency catheter ablation (RFCA) in patients with atrial fibrillation (AF).

**Methods:**

This retrospective multicenter study included 379 patients who underwent first-time RFCA and preprocedural CCTA at two centers (Center A, *n* = 238; Center B, *n* = 141). Patients from Center A were randomly divided into a training cohort (*n* = 166) and an internal validation cohort (*n* = 72) at a 7:3 ratio, whereas patients from Center B served as the external validation cohort. A TotalSegmentator model based on the nnU-Net v2 framework was used for fully automated segmentation of the left atrial body, left atrial appendage, and proximal pulmonary vein trunks, defined as the segments from the ostia to the first major bifurcations. These structures were merged and defined as the left atrial complex (LAC), which served as the unified region of interest for radiomics analysis. After assessing feature stability and reducing dimensionality, key features were selected to construct a radiomics score and an XGBoost-based radiomics model. A combined model was then developed by integrating the radiomics score with independent clinical predictors. Model performance was assessed using the area under the receiver operating characteristic curve (AUC), and model comparisons were performed using the DeLong test.

**Results:**

Of the 379 included patients, 106 (28.0%) developed late AF recurrence within 2 years after ablation. Multivariable logistic regression identified sex and left ventricular ejection fraction as independent clinical predictors. The combined model achieved AUCs of 0.896, 0.894, and 0.869 in the training, internal validation, and external validation cohorts, respectively, consistently outperforming the radiomics model (0.874, 0.840, and 0.787, respectively) and the clinical model (0.694, 0.658, and 0.704, respectively). These findings indicate good predictive performance, with maintained performance in the external validation cohort.

**Conclusion:**

The CCTA-based radiomics-clinical model derived from the LAC showed good performance for predicting late recurrence after RFCA for AF and may serve as a practical tool for individualized preprocedural risk stratification.

## Introduction

Atrial fibrillation (AF) is one of the most common arrhythmias in clinical practice (1, 2) and is strongly associated with increased risks of stroke, heart failure, and all-cause mortality (3). Current international guidelines recommend catheter ablation as a first-line therapy for symptomatic AF (4). However, its long-term efficacy remains limited by late recurrence (LR) after the procedure. The reported recurrence rate of AF within 1 year after ablation ranges from 20% to 40% and continues to increase with longer follow-up (5, 6). Therefore, accurate prediction of AF recurrence risk after ablation is clinically important for optimizing patient selection and guiding individualized management strategies.

Previous studies have established that clinical variables such as sex, AF type, left atrial diameter (LAD), left ventricular ejection fraction, and comorbidities are significantly associated with AF recurrence after catheter ablation (7, 8). Accordingly, clinical risk scores, including APPLE, CAAP-AF, DR-FLASH, and MB-LATER, have been developed for preprocedural risk stratification (9–12). However, these conventional clinical scores have shown limited discriminative ability for AF recurrence after ablation, with AUCs typically ranging from 0.55 to 0.67, suggesting that risk stratification based solely on clinical variables remains inadequate (13). Therefore, incorporating imaging information that reflects the risk of post-ablation AF recurrence may improve the accuracy of preprocedural risk stratification.

In recent years, increasing attention has been paid to the role of atrial structural remodeling in outcomes after catheter ablation for AF, with left atrial (LA) fibrosis recognized as a key determinant of ablation success (14). Late gadolinium enhancement cardiac magnetic resonance (LGE-CMR) enables noninvasive estimation of LA fibrotic burden based on the extent of enhancement, and this burden has been shown to be significantly associated with AF recurrence after ablation (15, 16). However, its routine clinical use remains limited by long acquisition times, high cost, and technical challenges in imaging the thin-walled atrium. Accordingly, a more accessible imaging strategy that can be readily incorporated into routine clinical workflows is needed.

In this context, radiomics has emerged as a promising analytic approach that enables the extraction of high-throughput quantitative features from medical images to characterize tissue heterogeneity beyond what visual assessment alone can provide. This technique has shown considerable potential for risk stratification and prognostic assessment in cardiovascular disease (17–19). Coronary computed tomography angiography (CCTA), a routine imaging examination in the preprocedural workup for AF ablation, not only provides coronary information but also clearly depicts the LA and surrounding structures, making it an attractive data source for radiomics analysis (20). Previous studies have used preprocedural CT to investigate the predictive value of several imaging features, including pulmonary vein-LA morphology, LA fractal geometry, and epicardial adipose tissue radiomics, for recurrence after AF ablation; however, their predictive performance has remained modest, with AUCs generally ranging from 0.68 to 0.81 (21–24). Moreover, most existing studies have relied on subjective, time-consuming manual segmentation and have either focused on a single anatomical region or analyzed periatrial tissues that only indirectly reflect atrial pathology, without fully integrating the interconnected anatomical structures involved in AF initiation and maintenance.

The initiation and maintenance of AF are governed not by a single anatomical structure but by the interplay between arrhythmic triggers and the substrate that sustains the arrhythmia. The pulmonary veins (PVs) are the predominant source of ectopic electrical activity, the LA body represents a key structural substrate for fibrosis, conduction slowing, and reentry, and the left atrial appendage (LAA) may serve as a non-PV trigger in a subset of patients (25–27). Previous studies have incorporated the LA body, PVs, and the left atrial wall into a unified fractal analysis framework and have shown that integrated models outperform single-structure models (28). However, most of these approaches still treated the LA as a relatively homogeneous entity. Notably, the LAA is distinct in its embryologic origin, anatomical configuration, and electrophysiological properties and should not be regarded merely as an appendage of the LA in the pathophysiology of AF. CT-based radiomics studies of the LAA-LA junction have shown that features derived from this region can discriminate patients with AF from controls, suggesting that the LAA-LA junction may provide information complementary to whole-LA analysis (29). Furthermore, a multicenter study reported that morphologic features of the proximal pulmonary vein trunks were significantly associated with post-ablation AF recurrence, whereas distal branches offered limited incremental predictive value (30). Notably, transitional zones between these structures, such as the PV-LA junction and LAA-LA ridge, represent critical interfaces for interactions between arrhythmogenic triggers and the substrate sustaining AF. Imaging information spanning these anatomical boundaries may not be fully captured when individual substructures are analyzed in isolation. Based on these observations, we defined the left atrial complex (LAC) as an integrated radiomics target by combining the LA body, LAA, and the bilateral superior and inferior pulmonary vein trunks into a single region of interest (ROI).

Accordingly, using preprocedural CCTA, we applied nnU-Net to segment the LAC automatically, extracted radiomics features from this region, and integrated them with clinical variables to develop a model to predict AF recurrence after RFCA. The model was further validated in an independent external cohort.

## Materials and methods

### Ethics statement

This retrospective study was approved by the Ethics Committees of Taixing People's Hospital (Approval No. LS2028024) and Jingjiang People's Hospital (Approval No. 2024-KY-013-01) and was conducted in accordance with the Declaration of Helsinki. The requirement for informed consent was waived because this study used retrospective, de-identified data.

### Study design and population

This multicenter retrospective study consecutively enrolled hospitalized patients who underwent first-time RFCA for non-valvular AF and completed preprocedural CCTA at Center A (Taixing People's Hospital) or Center B (Jingjiang People's Hospital) between January 2021 and April 2024. A total of 439 patients were initially assessed.

The inclusion criteria were as follows: (1) age ≥18 years, a confirmed diagnosis of paroxysmal or persistent AF, and undergoing first-time radiofrequency catheter ablation (RFCA); (2) CCTA performed within 1 week before ablation; (3) complete clinical data. The exclusion criteria were as follows: (1) moderate-to-severe valvular heart disease, hypertrophic cardiomyopathy, or congenital heart disease; (2) active malignancy or severe hepatic or renal dysfunction; and (3) CCTA images with severe motion artifacts, inadequate contrast opacification, or metal artifacts were excluded when image quality precluded reliable delineation of the LAC boundaries and subsequent analysis.

Ultimately, 406 patients met the eligibility criteria. All eligible patients were followed for up to 24 months after ablation, or until documented AF recurrence, whichever occurred first. During follow-up, 27 patients were not included in the final analysis because of relocation (*n* = 4), loss to follow-up (*n* = 18), or death (*n* = 5), leaving 379 patients in the final analytic cohort. The patient selection process is summarized in Figure 1.

**Figure 1:**
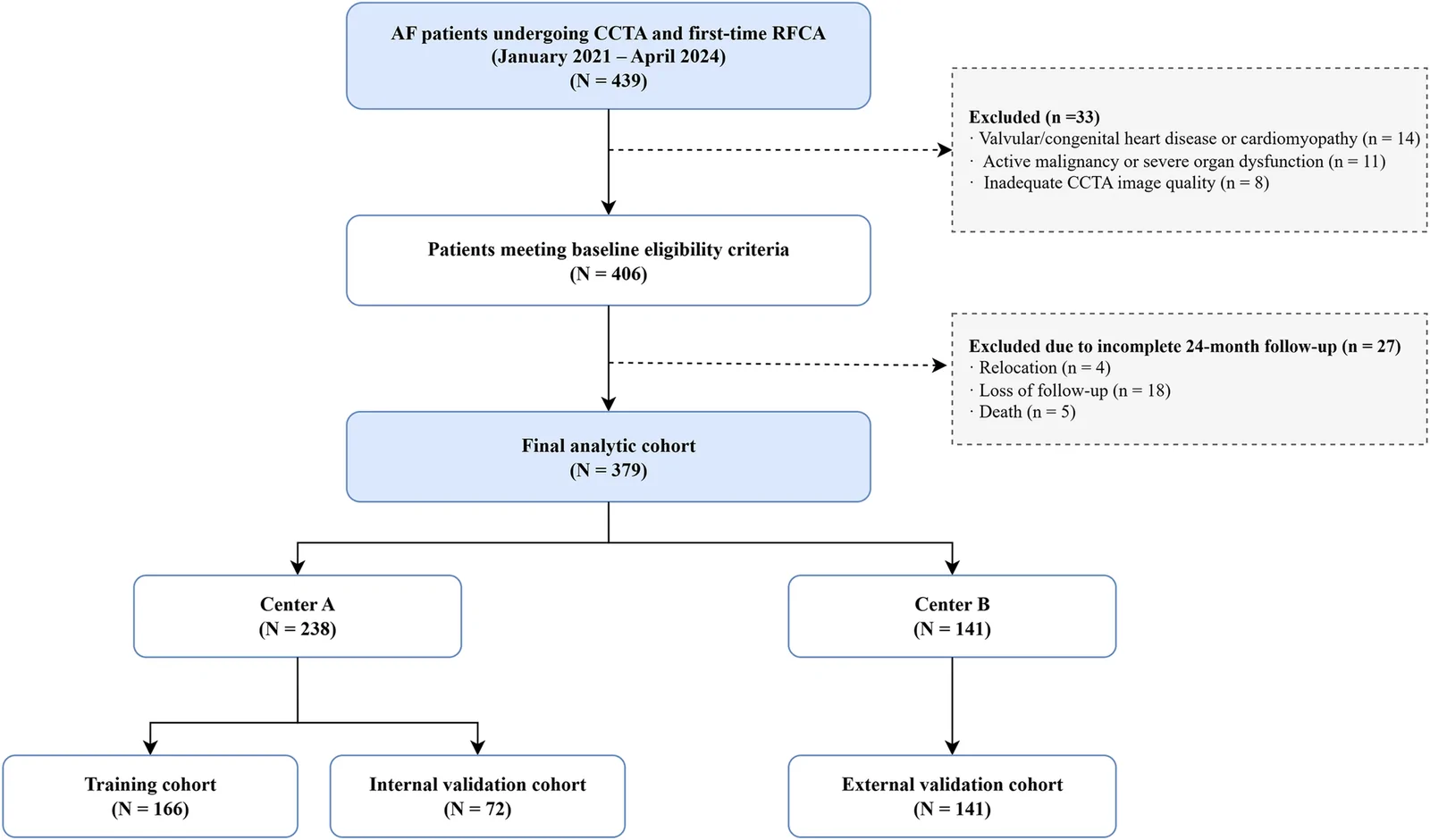
Flowchart of patient recruitment and study design. AF, atrial fibrillation; RFCA, radiofrequency catheter ablation; CCTA, coronary computed tomography angiography.

Among the 238 patients from Center A, 166 and 72 were randomly assigned to the training and internal validation cohorts, respectively, using a 7:3 split, whereas the 141 patients from Center B served as the external validation cohort.

Clinical data, including demographic characteristics, AF-related features, comorbidities, laboratory findings, echocardiographic parameters, and medication history, were extracted from the electronic medical record systems of both centers. Left ventricular ejection fraction (LVEF) was measured by transthoracic echocardiography using the biplane Simpson method (31).

### Ablation procedure

At both centers, radiofrequency catheter ablation was performed using the same CARTO 3 electroanatomical mapping system (Biosense Webster, Irvine, CA, USA). High-density LA endocardial mapping was performed using a PentaRay multipolar mapping catheter (Biosense Webster), and radiofrequency energy was delivered using a contact force-sensing, open-irrigated SmartTouch SF catheter (STSF; Biosense Webster). After double transseptal access, unfractionated heparin was administered intravenously at an initial dose of approximately 100 U/kg, with subsequent dosing adjusted according to intraoperative activated clotting time (ACT) measurements to maintain an ACT of 300–400 s. Ablation settings were guided by the Ablation Index (AI), with a target contact force of 10–30 g. Radiofrequency power was set at 40–50 W for the anterior wall and roof and 30–35 W for the posterior wall. The target AI values were 450–500 for the anterior wall, 380 for the posterior wall, and approximately 400 for the roof and inferior wall. Circumferential pulmonary vein isolation (PVI) was performed in all patients, followed by confirmation of bidirectional block and post-ablation remapping. In selected patients with persistent AF or abnormal atrial substrate identified during intraoperative electrophysiological assessment, additional ablation beyond PVI was performed according to intraoperative findings, including linear ablation, substrate modification, and superior vena cava isolation. If sinus rhythm was not restored after ablation, synchronized direct-current cardioversion at 150 J was performed under sedation, followed by repeat mapping to confirm the integrity of the ablation lines.

### Follow-up and endpoints

All patients were scheduled for outpatient follow-up at 3, 6, 12, 18, and 24 months after ablation. Each follow-up visit included symptom assessment and a 12-lead electrocardiogram (ECG), and 24-h Holter monitoring was performed at 3, 6, 12, and 24 months after ablation. Patients were instructed to seek prompt medical evaluation and undergo ECG recording if they developed symptoms such as palpitations, dyspnea, or chest tightness during follow-up. The primary endpoint was late atrial arrhythmia recurrence after RFCA, defined as any episode of AF, atrial flutter, or atrial tachycardia lasting >30 s documented by ECG or Holter monitoring beyond the 3-month post-ablation blanking period (32).

### CCTA acquisition protocol

At both centers, ECG-gated CCTA was performed using Siemens CT scanners. Center A used a dual-source CT scanner (SOMATOM Force), whereas Center B used a 128-slice CT scanner (SOMATOM Perspective). Before CCTA, oral metoprolol was administered to patients with a heart rate >70 beats/min and no contraindications to minimize the adverse effect of elevated heart rate on image quality. Nonionic iodinated contrast medium was administered via an antecubital vein. At Center A, acquisition timing was determined using a bolus-tracking technique with a trigger threshold of 100 HU, whereas a test-bolus technique was used at Center B. In both centers, acquisition timing was individualized to synchronize image acquisition with contrast arrival at the heart (33, 34), thereby minimizing interindividual variation in contrast enhancement associated with a fixed scan delay. Images were reconstructed at a slice thickness of ≤0.75 mm and exported for subsequent analysis. Detailed scanning parameters for both centers are provided in Supplementary Table S1.

### Definition of the LAC and ROI construction

The radiomics workflow is illustrated in Figure 2. In this study, the left atrial complex (LAC) was defined as a study-specific composite anatomical target comprising the left atrial body, the left atrial appendage, and the proximal trunk segments of the four pulmonary veins, extending from the ostia to the first major bifurcations. The LAC is not a standard anatomical term but a study-specific composite ROI constructed for the present radiomics analysis. Only the proximal pulmonary vein trunks were included because these segments are adjacent to the LA-PV junction, have relatively clear boundaries, and allow better segmentation consistency, whereas distal branches were excluded because their greater anatomical variability and smaller calibers may increase segmentation error and introduce feature noise (25, 30, 35).

**Figure 2:**
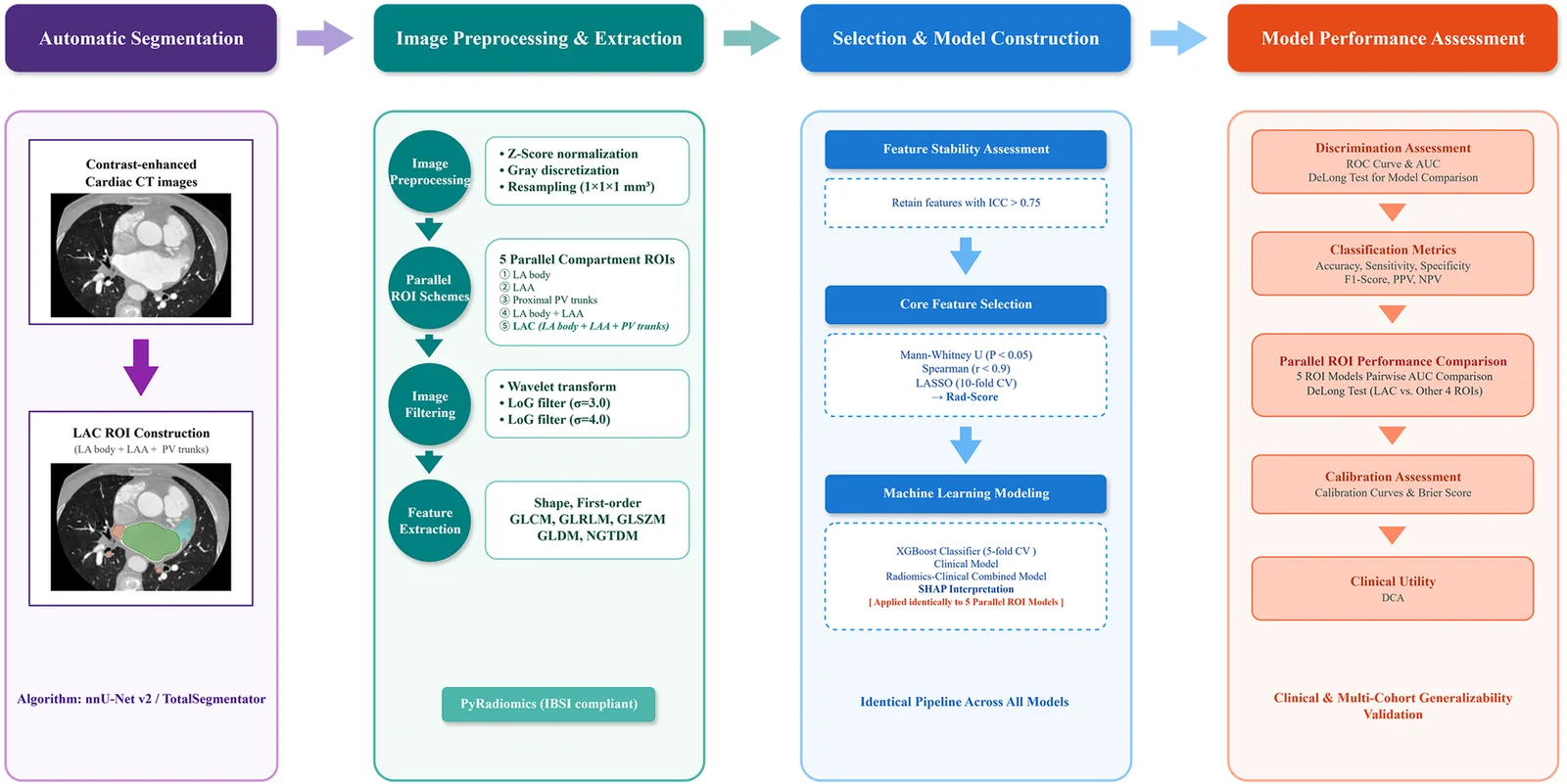
Flowchart of the current study workflow, demonstrating LAC-based CT radiomics for AF recurrence prediction. LAC, left atrial complex.

Given the differences in segmentation performance among substructures, the left atrial body, left atrial appendage, and proximal pulmonary vein trunks were segmented separately in a fully automated manner using TotalSegmentator and its derived pretrained models within the nnU-Net v2 framework (36, 37). The resulting three-dimensional segmentation masks were then merged in voxel space using a Boolean union operation to generate a unified LAC mask, which served as the ROI for radiomics feature extraction (37). Detailed boundary definitions, segmentation procedures, and model sources are provided in Supplementary Methods S1.

### Segmentation quality control and performance evaluation

All automated segmentations underwent manual quality review. The segmentations were independently visually reviewed by two radiologists, each with more than 10 years of experience in cardiovascular imaging, using 3D Slicer (version 5.2.1) to assess the agreement between the segmentation boundaries and the corresponding anatomical structures. In cases of disagreement, a third, more senior radiologist adjudicated the discrepancy. Cases with unacceptable segmentation quality were excluded. The detailed workflow is shown in Figure 1. Cases that passed quality review were accepted without manual correction.

To quantitatively assess automated segmentation performance, the Dice coefficients achieved by each segmentation model on its respective original validation set are summarized in Supplementary Table S2A. In addition, 30 patients were randomly sampled from the overall cohort for independent validation against manually created reference segmentations. For the three constituent regions of the LAC—the LA body, LAA, and proximal PV trunks—a radiologist with >10 years of experience in cardiovascular imaging generated reference segmentations slice by slice using nnInteractive (38). A second radiologist with comparable experience reviewed and verified all segmentations, and any discrepancies were resolved by consensus. Dice coefficients and intersection-over-union (IoU) values comparing the automated and reference segmentations are presented in Supplementary Table S2B.

### Image preprocessing and radiomic feature extraction

Radiomic feature extraction was performed in accordance with the Image Biomarker Standardization Initiative (IBSI) guidelines (39). To ensure robust radiomic feature extraction, all contrast-enhanced cardiac CT images and their corresponding LAC masks underwent standardized preprocessing. The principal preprocessing steps were as follows. Images were first resampled to an isotropic voxel spacing of 1 mm^3^ × 1 mm^3^ × 1 mm^3^ to standardize spatial resolution across scans. Image intensities were then normalized using *Z*-score normalization to reduce interscan differences in overall intensity level and distribution. Finally, gray-level discretization was performed using a fixed bin width of 25 (binWidth = 25) for subsequent texture feature extraction. In addition, wavelet decomposition and Laplacian-of-Gaussian filtering (*σ* = 3.0 and 4.0) were applied to the preprocessed images to capture multiscale textural information. CT radiomic features were then extracted using the PyRadiomics package in Python, including shape, first-order, and texture features derived from the gray-level co-occurrence matrix, gray-level run-length matrix, gray-level size zone matrix, gray-level dependence matrix, and neighboring gray-tone difference matrix.

### Assessment of radiomic feature robustness

To ensure feature stability, a robustness screening step was performed after feature extraction. Although a fully automated segmentation algorithm was used, algorithm-related uncertainty and the robustness of radiomic features to minor segmentation variations were assessed by randomly selecting 30 cases from the training cohort and repeating the segmentation pipeline 10 times with different random seeds. For each feature, the intraclass correlation coefficient (ICC) was calculated across the 10 repeated measurements using a two-way random-effects model with absolute agreement. Features with an ICC > 0.75 were considered sufficiently stable and were retained for subsequent feature selection and analysis.

### Radiomic feature selection and rad-score construction

A multi-step feature reduction and selection pipeline was applied in the training cohort. First, the Mann–Whitney *U* test was used for initial univariable screening of all CCTA radiomic features, and features with *P* < 0.05 were retained. To reduce redundancy among the retained features, pairwise correlations were evaluated using Spearman's rank correlation coefficient. When the absolute correlation coefficient between any two features exceeded 0.9, an iterative greedy elimination strategy was applied, whereby the most redundant feature in the current set was removed at each step. Subsequently, LASSO regression was performed to identify the most predictive subset of features. The penalty parameter was determined by 10-fold cross-validation, and features with non-zero coefficients were retained. All the above analyses were performed using the scikit-learn package in Python (version 1.0.1). The Rad-score for each patient was then calculated as a weighted linear combination of the retained features and their corresponding coefficients.

### Model building

The radiomic features retained after LASSO selection were used to develop an XGBoost-based radiomics model to evaluate their predictive value for post-ablation AF recurrence. The model was developed in the training cohort, and hyperparameter optimization was performed using 5-fold cross-validation, with the mean cross-validated AUC used as the primary performance metric. To reduce overfitting and improve generalizability, the final model was configured with the following key hyperparameters: n_estimators = 100, learning_rate = 0.05, and max_depth = 4, thereby limiting the complexity of individual trees. In addition, random subsampling was applied to both samples and features, with subsample = 0.8 and colsample_bytree = 0.8. The L1 and L2 regularization parameters were set to reg_alpha = 0.1 and reg_lambda = 1.0, respectively, to further constrain model complexity. To improve the interpretability of the XGBoost model, Shapley Additive Explanations (SHAP) values were used to quantify the direction and magnitude of each radiomic feature's contribution to the model's output.

Univariable and multivariable logistic regression analyses were performed for the Rad-score and clinical variables to identify independent predictors of post-ablation AF recurrence. A clinical model was developed using the independent clinical predictors, whereas the radiomics model was established using the XGBoost algorithm. To facilitate clinical implementation and nomogram-based visualization, the Rad-score was further integrated with the independent clinical predictors to develop a combined radiomics-clinical model. All models were developed and optimized in the training cohort, subsequently evaluated in the internal validation cohort, and further validated in an independent external cohort to assess their robustness and generalizability.

### Model performance evaluation

Model performance was assessed using receiver operating characteristic (ROC) curves. The area under the curve (AUC), accuracy, sensitivity, specificity, F1 score, positive predictive value (PPV), and negative predictive value (NPV) were calculated for each model. Differences in AUCs between models were compared using the DeLong test. Calibration was assessed using calibration curves and the Brier score, whereas clinical net benefit across a range of threshold probabilities was evaluated using decision curve analysis (DCA).

### Comparison of region-of-interest strategies

To evaluate the predictive performance of LAC relative to its constituent subregions and the conventional whole-LA ROI, five parallel ROIs were defined using the previously generated automated segmentation masks of the LA body, LAA, and proximal PV trunks: the LA body, LAA, proximal PV trunks, LA body + LAA (representing the conventional whole-LA ROI), and LAC (LA body + LAA + proximal PV trunks). All ROIs underwent identical image preprocessing, feature extraction, feature selection, and model development procedures and were evaluated in parallel using the same training, internal validation, and external validation cohorts. Predictive performance was assessed using the AUC, and pairwise differences in AUC between the LAC model and each of the other ROI-based models were evaluated in the external validation cohort using the DeLong test.

### Statistical analysis

Statistical analyses and model development were performed using SPSS (version 26.0), R (version 4.5.1, https://www.R-project.org), and Python (version 3.8). Continuous variables were first assessed for normality using the Shapiro–Wilk test. Normally distributed variables were expressed as mean ± standard deviation (SD) and compared using the independent-samples *t* test or one-way analysis of variance, as appropriate. Non-normally distributed variables were expressed as median [interquartile range (IQR)] and compared using the Mann–Whitney *U* test or the Kruskal–Wallis *H* test, as appropriate. Categorical variables were expressed as counts (percentages) and compared using the chi-square test; Fisher's exact test was used when the expected cell count was <5. All tests were two-sided, and *P* < 0.05 was considered statistically significant.

## Results

### Patient characteristics

The baseline characteristics of the three cohorts are summarized in Table 1. Significant differences were observed across the three cohorts in sex (*P* = 0.008), BMI (*P* = 0.026), and LVEF (*P* < 0.001), whereas the remaining baseline characteristics did not differ significantly among the cohorts (all *P* > 0.05).

**Table 1:** Baseline characteristics of patients in the training, internal test, and external validation cohorts.

Characteristics		Training cohort (*n* = 166)	Internal validation cohort (*n* = 72)	External validation cohort (*n* = 141)	*P*
Age (years)		68.0 [59.0, 72.0]	67.0 [58.0, 72.2]	69.0 [63.0, 75.0]	0.060
Sex, *n* (%)	Female	82 (49.4)	23 (31.9)	49 (34.8)	0.008
	Male	84 (50.6)	49 (68.1)	92 (65.2)	
BMI (kg/m²)		23.6 [22.5, 25.4]	24.1 [21.5, 26.0]	24.7 [22.9, 27.0]	0.026
AF type, *n* (%)	PAF	117 (70.5)	52 (72.2)	88 (62.4)	0.216
	PeAF	49 (29.5)	20 (27.8)	53 (37.6)	
AF duration (months)		12.0 [1.0, 36.0]	24.0 [1.0, 48.0]	12.0 [1.0, 24.0]	0.152
CHF, *n* (%)		39 (23.5)	19 (26.4)	33 (23.4)	0.871
Hypertension, *n* (%)		94 (56.6)	37 (51.4)	63 (44.7)	0.113
Diabetes, *n* (%)		35 (21.1)	13 (18.1)	26 (18.4)	0.794
Prior stroke, *n* (%)		24 (14.5)	10 (13.9)	18 (12.8)	0.911
Smoking, *n* (%)		42 (25.3)	19 (26.4)	40 (28.4)	0.831
Alcohol, *n* (%)		31 (18.7)	15 (20.8)	29 (20.6)	0.890
LAD (mm)		42.0 [38.0, 46.0]	41.5 [38.8, 45.0]	42.2 [39.0, 46.0]	0.785
LVEF (%)		60.0 [57.0, 63.0]	61.0 [56.8, 64.0]	62.0 [59.0, 68.0]	<0.001
NLR		2.3 [1.8, 3.4]	2.4 [1.8, 3.7]	2.1 [1.6, 3.4]	0.189
Amiodarone, *n* (%)		95 (57.2)	39 (54.2)	74 (52.5)	0.700
Beta-blocker, *n* (%)		60 (36.1)	28 (38.9)	44 (31.2)	0.481
CCB, *n* (%)		52 (31.3)	22 (30.6)	30 (21.3)	0.117

AF, atrial fibrillation; PAF, paroxysmal atrial fibrillation; PeAF, persistent atrial fibrillation; BMI, body mass index; CHF, congestive heart failure; LAD, left atrial diameter; LVEF, left ventricular ejection fraction; NLR, neutrophil-to-lymphocyte ratio; CCB, calcium channel blocker.

Data are presented as median [Q1, Q3] or *n* (%). *P* values were calculated using the Kruskal–Wallis *H* test for continuous variables and Pearson's chi-square test or Fisher's exact test for categorical variables, as appropriate.

During the 24-month follow-up, 106 patients (28.0%) developed LR. The rates of LR were 32.5% (54/166), 31.9% (23/72), and 20.6% (29/141) in the training, internal validation, and external validation cohorts, respectively.

The characteristics and predictors of patients with and without LR in the training cohort are summarized in Table 2. In univariable logistic regression analysis, male sex (OR = 0.393, 95% CI: 0.200–0.769, *P* < 0.001), LAD (OR = 1.07, *P* = 0.029), LVEF (OR = 0.95, *P* = 0.028), CHF (OR = 2.10, *P* = 0.042), and the Rad-score were significantly associated with LR. In multivariable logistic regression, sex, LVEF, and the Rad-score remained independent predictors of LR. Therefore, sex (*P* < 0.001) and LVEF (*P* = 0.011) were incorporated into the clinical model, while the Rad-score was further used to construct the combined model.

**Table 2:** Characteristics and predictors of late recurrence in the training cohort.

Variables	No recurrence (*n* = 112)	Recurrence (*n* = 54)	Univariable LR			Multivariable LR		
			OR	95% CI	*P*	OR	95% CI	*P*
Age (years)	68.0 [59.8, 72.2]	67.5 [57.2, 71.8]	1.011	0.971–1.044	0.704			
Sex (male)	65 (58.0%)	19 (35.2%)	0.393	0.200–0.769	0.007	0.290	0.144–0.582	＜0.001
BMI (kg/m²)	23.6 [22.5, 25.4]	23.9 [22.8, 25.7]	0.980	0.880–1.090	0.719			
AF type (persistent)	27 (24.1%)	22 （40.7%)	1.890	0.933–3.824	0.077			
AF duration (months)	6.0 [0.4, 24.2]	12.0 [2.2, 42.8]	1.003	0.995–1.011	0.453			
CHF	19 (17.0%)	20 (37.0%)	2.101	1.029–4.292	0.042	1.218	0.517–2.873	0.652
Hypertension	60 (53.6%)	34 (63.0%)	1.071	0.560–2.050	0.836			
Diabetes	29 (25.9%)	6 (11.1%)	0.870	0.380–1.991	0.741			
Prior stroke	15 (13.4%)	9 (16.7%)	2.035	0.860–4.817	0.106			
Smoking	34 (30.4%)	8 (14.8%)	0.447	0.196–1.016	0.055			
Alcohol	23 (20.5%)	8 (14.8%)	0.619	0.268–1.430	0.261			
LAD (mm)	42.0 [38.0,46.0]	43.5 [41.0,47.8]	1.070	1.007–1.138	0.029	1.043	0.973–1.118	0.232
LVEF (%)	60.0 [57.0, 64.0]	58.0 [56.0, 62.0]	0.947	0.902–0.994	0.028	0.955	0.902–0.998	0.011
NLR	2.3 [1.7, 3.5]	2.3 [1.9, 3.0]	1.035	0.927–1.156	0.540			
Amiodarone	56 (50.0%)	39 (72.2%)	1.736	0.895–3.366	0.103			
Beta-blocker	44 (39.3%)	16 (29.6%)	0.629	0.317–1.248	0.185			
CCB	35 (31.2%)	17 (31.5%)	0.658	0.319–1.355	0.256			
Rad-score	0.3 (0.1)	0.4 (0.2)	1.006	1.003–1.009	<0.001	1.128	1.043–1.218	0.002

AF, atrial fibrillation; BMI, body mass index; CHF, congestive heart failure; LAD, left atrial diameter; LVEF, left ventricular ejection fraction; NLR, neutrophil-to-lymphocyte ratio; CCB, calcium channel blocker; Rad-score, radiomics score; OR, odds ratio; CI, confidence interval.

Data are presented as median [Q1, Q3] or *n* (%). For sex, female was used as the reference category.

### Radiomics feature selection and model interpretation

Among the 1,050 radiomic features extracted from the LAC, 1,029 with ICC > 0.75 were retained for subsequent analysis. After Mann–Whitney *U* test-based univariable screening, 429 candidate features with *P* < 0.05 were retained. After Spearman correlation filtering to remove highly redundant features, 129 features remained. Final feature selection using LASSO regression (optimal *λ*min = 0.0579; Figure 3) identified six core radiomic features with non-zero coefficients. To further evaluate correlations among the final selected features, a hierarchical clustering correlation heatmap was constructed (Supplementary Figure S1). All six selected features were wavelet-derived first-order or texture features, with no shape features retained. These features primarily characterized intensity distribution, intensity variability, and textural heterogeneity within LAC rather than directly quantifying morphologic characteristics such as LA volume or wall thickness. Detailed definitions of the individual features and their directions of association with AF recurrence are provided in Supplementary Table S3.

**Figure 3:**
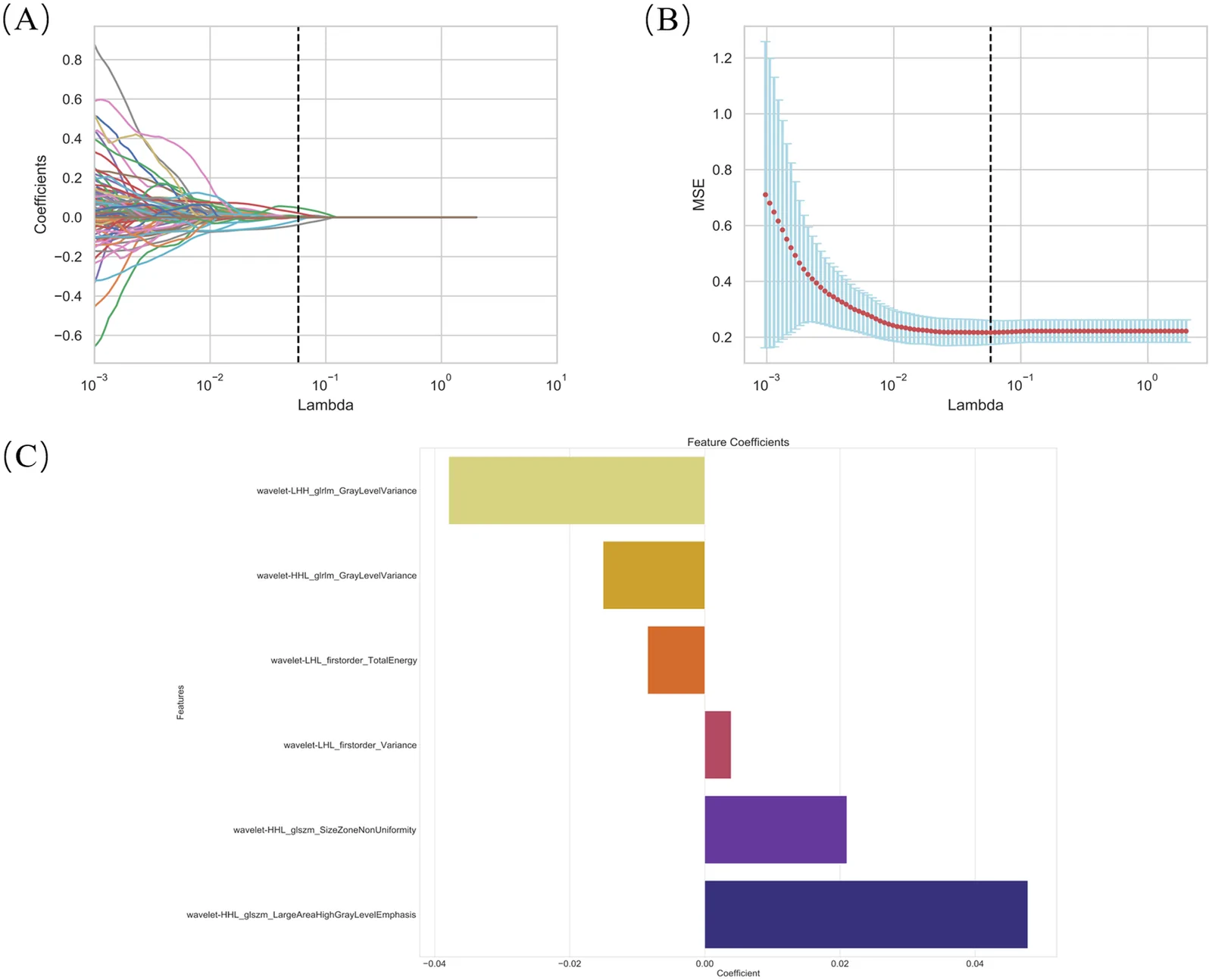
**(A)** LASSO regression coefficient of the features. **(B)** The most valuable features were screened out by tuning Lambda using LASSO via minimum binomial deviation. **(C)** The 6 final retained radiomic features and their corresponding weight coefficients. LASSO, Least Absolute Shrinkage and Selection Operator.

Based on these six features and their corresponding LASSO coefficients, a Rad-score was constructed, with the formula provided in Supplementary Methods S2. As shown in Table 2, the Rad-score was significantly higher in patients with LR than in those without LR in the training cohort [0.58 (0.32, 0.81) vs. 0.21 (0.08, 0.39), *P* < 0.001].

An XGBoost-based radiomics model was then developed using the selected radiomic features. To interpret the model, SHAP analysis was performed to quantify each feature's contribution to the model's output (Figure 4). The three features with the greatest contribution to model prediction were wavelet-LHL_firstorder_TotalEnergy, wavelet-LHL_firstorder_Variance, and wavelet-HHL_glszm_SizeZoneNonUniformity. Higher values of wavelet-LHL_firstorder_Variance and wavelet-HHL_glszm_SizeZoneNonUniformity were mainly associated with positive SHAP values, whereas higher values of wavelet-LHL_firstorder_TotalEnergy were mainly associated with negative SHAP values, suggesting that greater gray-level variability and textural heterogeneity were associated with a higher predicted risk of AF recurrence, whereas higher total energy was associated with a lower predicted risk.

**Figure 4:**
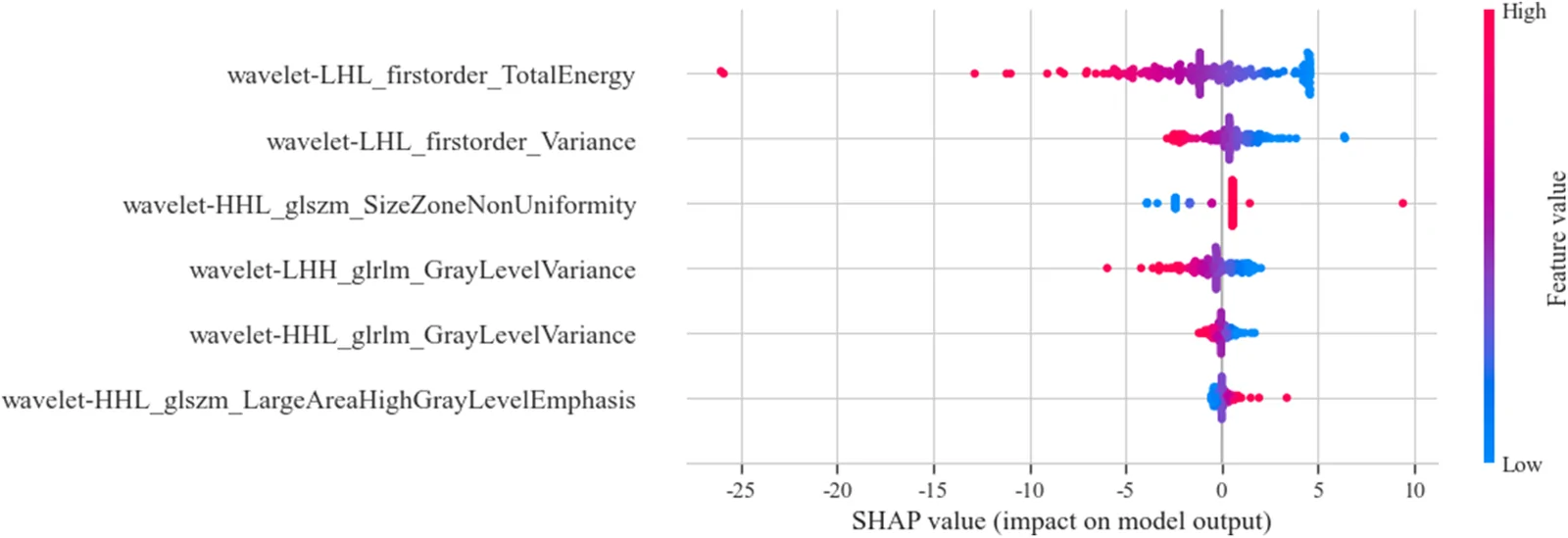
SHAP-based interpretation of the combined model, showing the contribution of individual features to model predictions. SHAP, Shapley additive explanations.

### Development and performance of the predictive models

Three predictive models were developed in this study: a clinical model incorporating sex and LVEF; an XGBoost-based radiomics model using the selected radiomic features; and a combined radiomics-clinical model integrating the Rad-score, sex, and LVEF. Figure 5 shows the nomogram derived from the combined model for individualized estimation of recurrence risk. Table 3 summarizes the predictive performance of the three models across the training, internal validation, and external validation cohorts, and Figure 6 presents the corresponding receiver operating characteristic (ROC) curves. The combined model achieved AUCs of 0.896 (95% CI: 0.859–0.930), 0.894 (95% CI: 0.816–0.961), and 0.869 (95% CI: 0.811–0.921), respectively. The corresponding AUCs were 0.694, 0.658, and 0.704 for the clinical model and 0.874, 0.840, and 0.787 for the radiomics model. Across all three cohorts, the combined model consistently showed higher AUCs than the other two models. In the internal validation cohort, DeLong's test showed that the combined model had a significantly higher AUC than the clinical model (*P* = 0.032) and the radiomics model (*P* = 0.014) (Table 4). In the external validation cohort, the combined model achieved a sensitivity of 0.741 and a specificity of 0.920, suggesting good generalizability.

**Figure 5:**
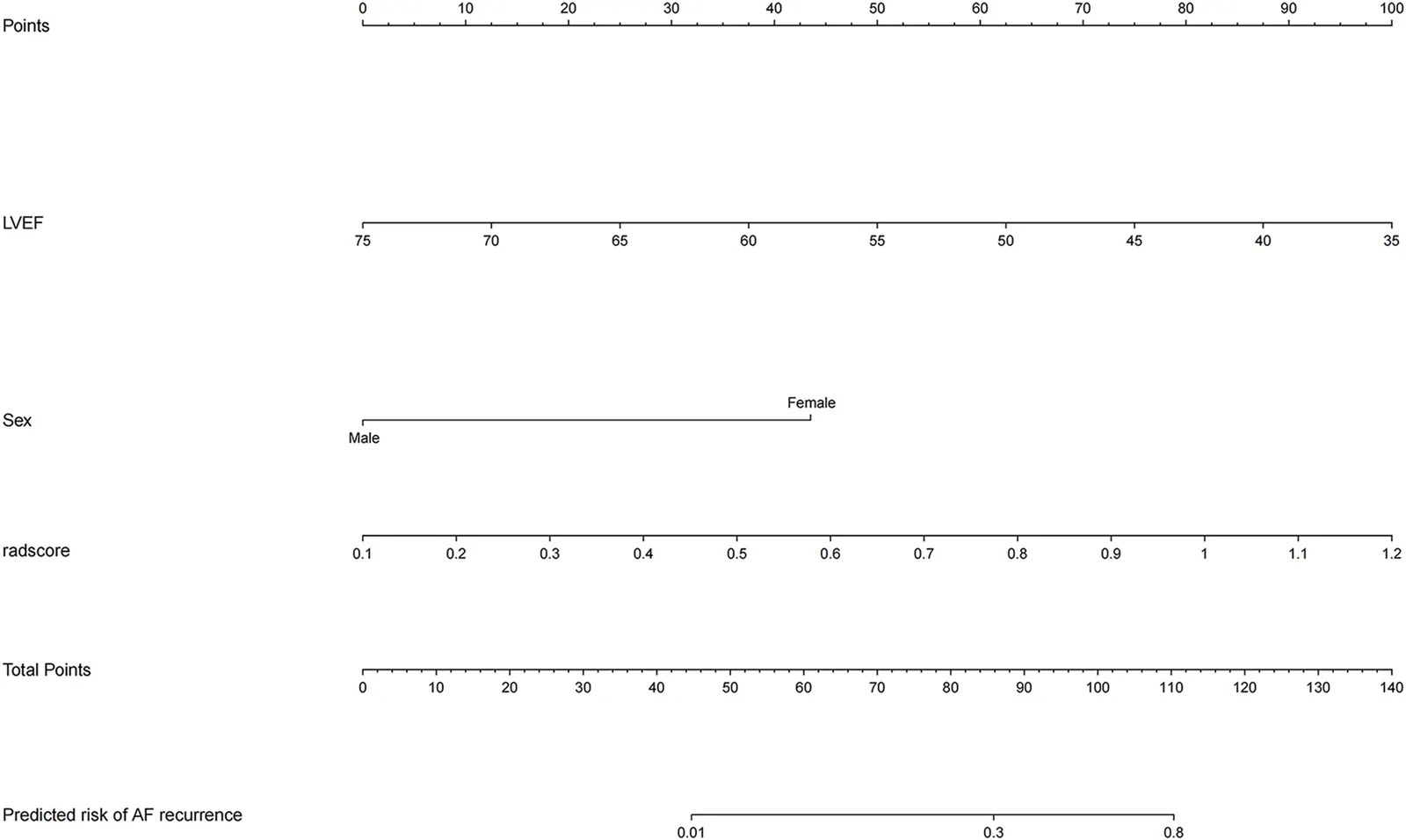
The nomogram of a predictive model combining radiomics and clinical features.

**Table 3:** Model performance for predicting late recurrence after AF ablation.

Cohort	Model	AUC	95% CI	Accuracy	Sensitivity	Specificity	F1-score	PPV	NPV
Training cohort	Clinical Model	0.694	0.630, 0.758	0.655	0.740	0.615	0.582	0.479	0.832
	Radiomics Model	0.874	0.834, 0.910	0.798	0.831	0.783	0.727	0.646	0.906
	Combined Model	0.896	0.859, 0.930	0.857	0.805	0.882	0.785	0.765	0.904
Internal validation cohort	Clinical Model	0.658	0.535, 0.771	0.653	0.652	0.653	0.545	0.469	0.800
	Radiomics Model	0.840	0.760, 0.915	0.764	0.783	0.755	0.679	0.600	0.881
	Combined Model	0.894	0.816, 0.961	0.875	0.783	0.918	0.800	0.818	0.900
External validation cohort	Clinical Model	0.704	0.630, 0.774	0.705	0.500	0.804	0.524	0.551	0.769
	Radiomics Model	0.787	0.723, 0.845	0.675	0.852	0.589	0.630	0.500	0.892
	Combined Model	0.869	0.811, 0.921	0.861	0.741	0.920	0.777	0.816	0.880

PPV, positive predictive value; NPV, negative predictive value.

**Figure 6:**
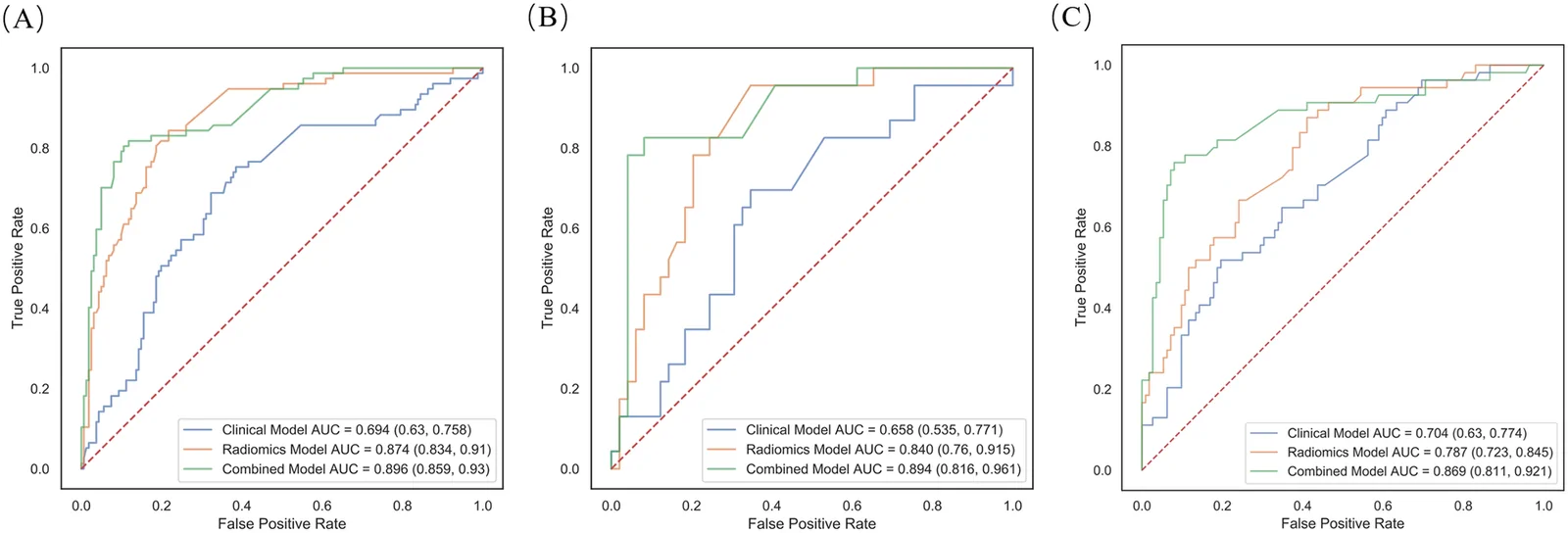
**(A)** ROC curves of the three models in the training set. **(B)** ROC curves of the three models in the internal validation set. **(C)** ROC curves of the three models in the external validation set. ROC, receiver operating characteristic.

**Table 4:** Comparison of model performance using deLong's test in the internal validation cohort.

Comparison of predictive models	Difference between AUCs	*P*
Combined vs. Radiomic	0.054	0.014
Combined vs. Clinical	0.236	0.032
Radiomic vs. Clinical	0.182	0.046

AUC, area under the curve.

### Comparison of predictive performance between LAC and other ROI schemes

The LAC model achieved the highest AUCs across the training, internal validation, and external validation cohorts, with values of 0.874, 0.840, and 0.787, respectively, followed by the LA body + LAA ROI (0.839/0.756/0.780), LA body (0.761/0.686/0.656), LAA (0.728/0.659/0.610), and proximal PV trunks (0.674/0.622/0.590) (Figure 7).

**Figure 7:**
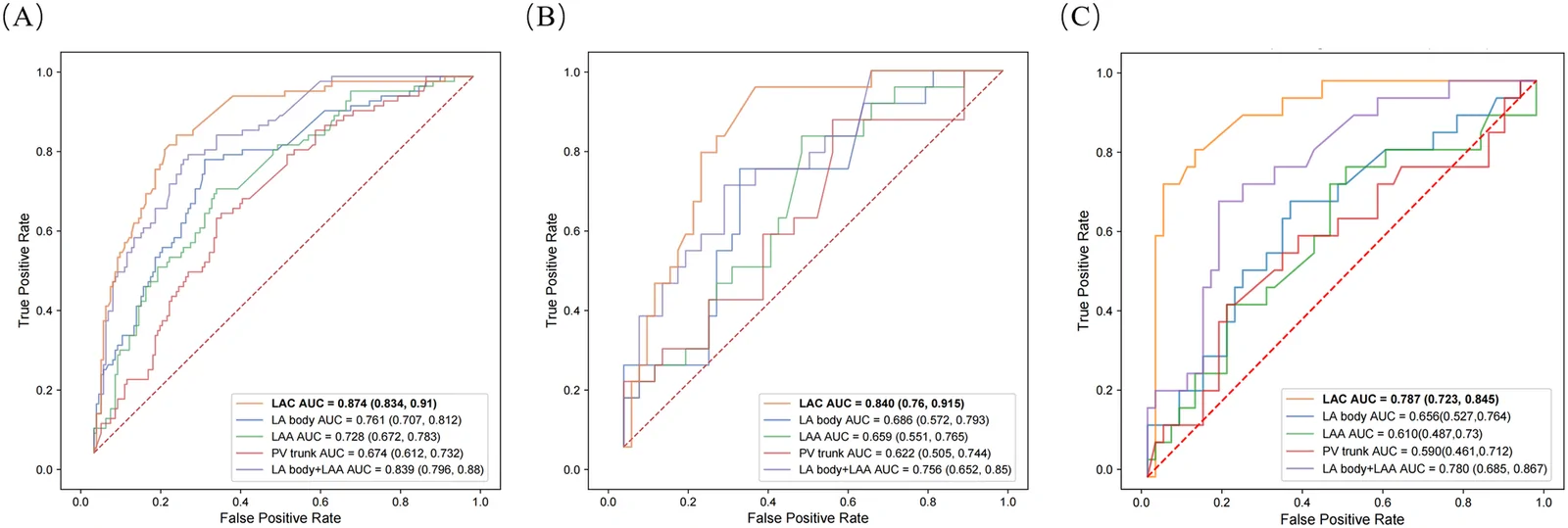
**(A)** ROC curves for the five ROI models in the training set. **(B)** ROC curves for the five ROI models in the internal validation set. **(C)** ROC curves for the five ROI models in the external validation set. ROI, region of interest; LA body, left atrial body; LAA, left atrial appendage; PV trunk, pulmonary vein trunk.

In the external validation cohort, DeLong's test showed that the AUC of the LAC model was significantly higher than those of the LAA (*P* = 0.024), proximal PV trunks (*P* = 0.008), and LA body + LAA ROI (*P* = 0.027) models, whereas the difference between the LAC and LA body models was not statistically significant (*P* = 0.314) (Supplementary Figure S2).

### Calibration and clinical utility

Figure 8 shows good agreement between the predicted probabilities and the observed rates of LR after AF ablation across the training, internal test, and external validation cohorts. The corresponding Brier scores were 0.083, 0.082, and 0.124, indicating good overall probabilistic performance. Figure 9 shows that the combined model provided greater net clinical benefit for predicting LR after AF ablation.

**Figure 8:**
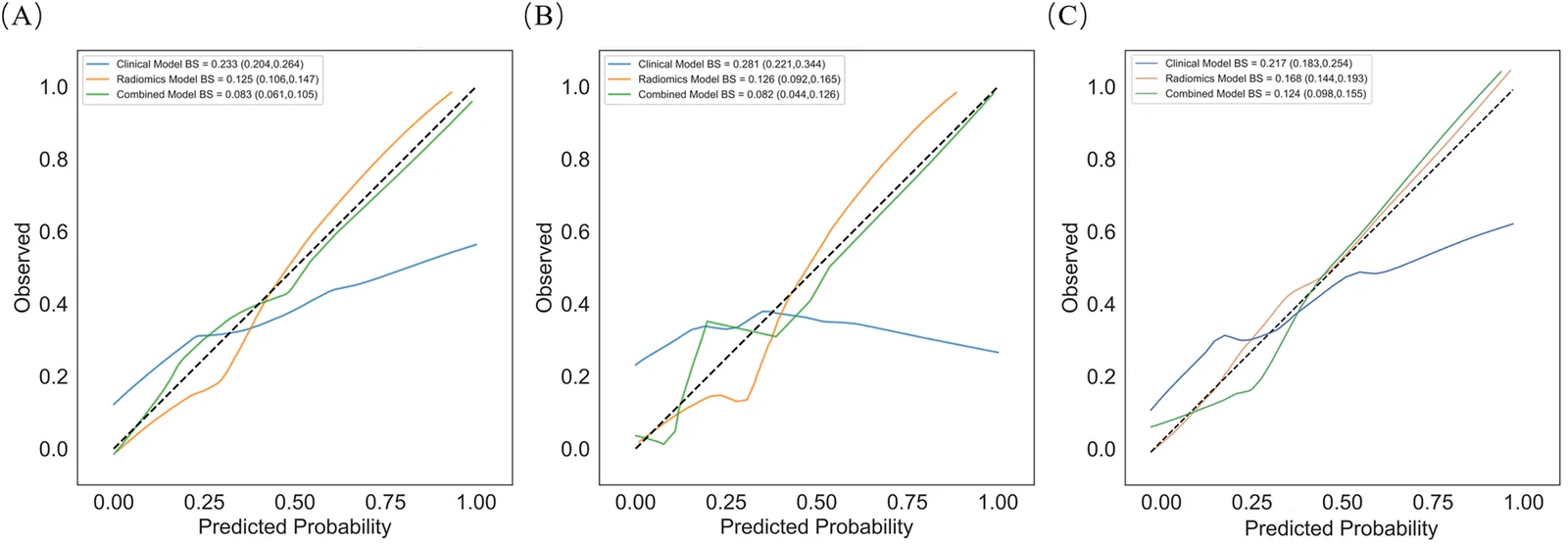
**(A)** calibration curves and brier scores for the three models in the training set. **(B)** Calibration curves and Brier scores for the three models in the internal validation set. **(C)** Calibration curves and Brier scores for the three models in the external validation set.

**Figure 9:**
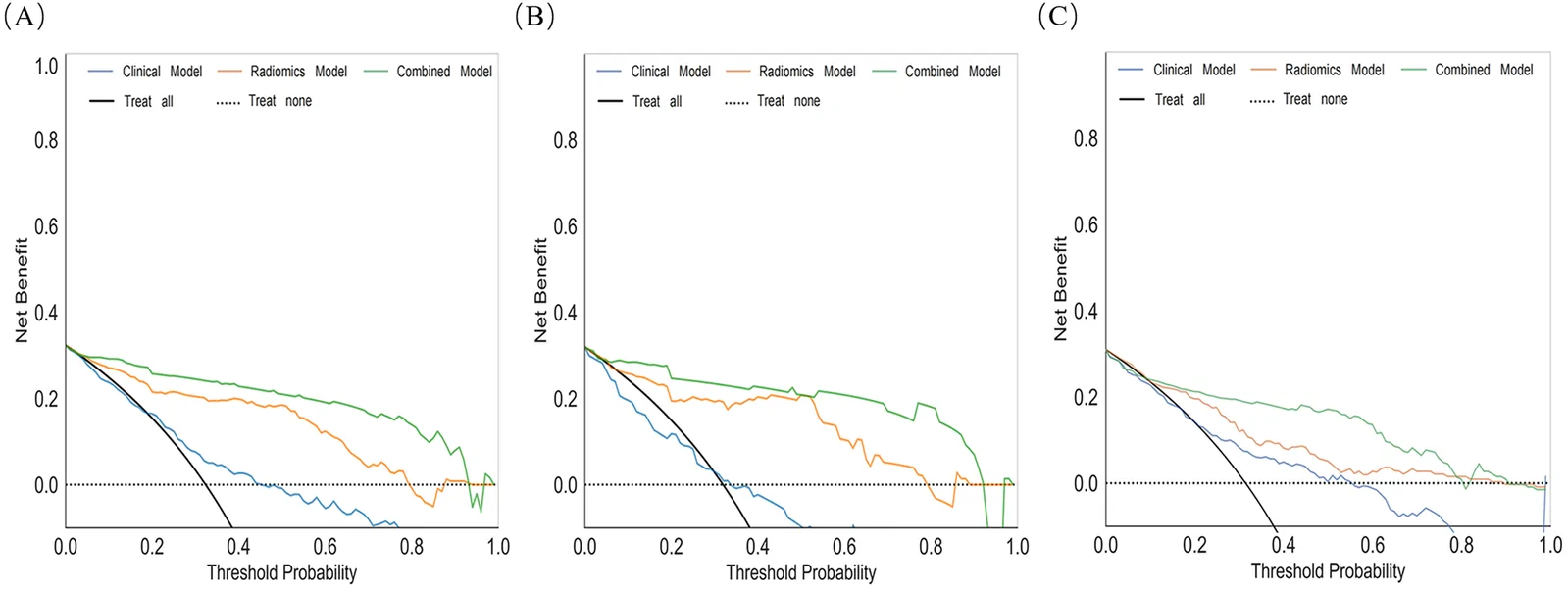
**(A)** DCA curves of the three models in the training set. **(B)** DCA curves of the three models in the internal validation set. **(C)** DCA curves of the three models in the external validation set. DCA, decision curve analysis.

## Discussion

We developed and externally validated a fully automated radiomics-clinical model based on routine CCTA to predict LR after catheter ablation in patients with AF. In the external validation cohort, the model showed good discrimination (AUC = 0.869), and the corresponding nomogram provided a practical, noninvasive tool for preprocedural risk stratification.

The comparatively favorable performance of the LAC model may be attributable, at least in part, to its ROI design. Post-ablation AF recurrence reflects the combined effects of arrhythmogenic triggers and the substrate that sustains AF. PVs are the predominant source of AF triggers, whereas the LAA can serve as a non-PV trigger in some patients; diffuse fibrotic and electrically remodeled substrate throughout the LA contributes to AF maintenance (25–27). These anatomical components implicated in AF initiation and maintenance are contiguous, and their intracavitary flow patterns and contrast distribution are spatially coupled. Prior studies have shown that anatomical transition zones, such as the LAA-LA junction, contain radiomic information independently associated with AF (29). Analyzing these substructures separately may therefore fail to capture spatial information spanning their boundaries.

Accordingly, we combined the LA body, LAA, and proximal PV trunks into a single LAC ROI to cover a broader anatomical domain relevant to both AF triggers and substrate, while preserving spatial heterogeneity across anatomical transition zones within a standardized segmentation. This design is consistent with previous studies showing complementary predictive information from multiple cardiac structures (28, 30), as well as predictive value from integrated CT analysis encompassing the LA and proximal PVs (40). In addition, combined analysis of LA and PV morphology using geometric and fractal features has demonstrated the potential value of integrating information across multiple atrial structures (41).

To assess the incremental value of ROI coverage while holding the remaining modeling procedures constant, we constructed five parallel models that differed only in ROI extent; data partitioning, image preprocessing, feature extraction and selection, and classification were otherwise identical. As shown in Figure 7 and Supplementary Figure S2, the LAC model achieved the highest discriminative performance numerically in the external validation cohort. DeLong testing showed that its AUC was significantly higher than those of the LAA model (*P* = 0.024), PV model (*P* = 0.008), and LA body + LAA model (*P* = 0.027), whereas the difference from the LA body model was not statistically significant (*P* = 0.314). These findings suggest that inclusion of the proximal PV trunks may contribute additional predictive information; however, the lack of a significant difference from the LA body model indicates that the dominant predictive signal may still reside within the LA body.

Because these ROI comparisons were exploratory and the sample size was relatively limited, the findings should be interpreted cautiously. They do not establish the superiority of the LAC strategy and require confirmation in larger independent cohorts. In addition, compared with previous approaches requiring separate manual delineation of multiple regions (23, 24, 42), the fully automated nnU-Net-based LAC framework may reduce operator dependence and interobserver variability. The radiomics model achieved an AUC of 0.787 in the multicenter external validation cohort, providing preliminary support for its generalizability across different CT scanners and acquisition protocols. Prospective multicenter validation is nevertheless warranted.

Based on the final selected features and the SHAP analysis (Figure 4), all six retained features were derived from wavelet-filtered images and comprised both first-order statistical and higher-order texture features. Their mathematical definitions, SHAP directions, and potential clinical implications are detailed in Supplementary Table S3, together with the nomenclature of the wavelet sub-bands. By definition, these features capture three broad aspects of image information: first-order gray-level energy and dispersion, GLRLM-based run-length heterogeneity, and GLSZM-based heterogeneity in gray-level zone-size patterns. These findings suggest that imaging information associated with LR after AF ablation may extend beyond macroscopic morphological measures such as LA volume or wall thickness and may also relate to the multiscale distribution patterns and textural heterogeneity of CT gray-level signals within the LAC.

Of note, the radiomic features analyzed in this study were extracted from the contrast-enhanced LAC blood pool on CCTA rather than from the atrial wall. Gray-level patterns within the LAC blood pool may indirectly reflect atrial structural and functional alterations through several mechanisms, including geometric remodeling such as AF-related LA enlargement and changes in LAA morphology (43), partial-volume effects at the blood pool-myocardial interface caused by limited spatial resolution, and hemodynamics-related differences in intracavitary contrast distribution. However, these links have not been directly validated. Accordingly, the mechanistic interpretations below should be considered hypothesis-generating rather than direct evidence of atrial wall histopathology.

SHAP analysis showed that higher wavelet-LHL_firstorder_Variance, wavelet-HHL_glszm_SizeZoneNonUniformity, and wavelet-LHH_glrlm_GrayLevelVariance values were generally associated with a higher model-predicted likelihood of recurrence. These features respectively reflect gray-level dispersion, nonuniformity in zone-size distribution, and gray-level variability across runs. The joint retention of GLRLM-GrayLevelVariance in both the LHH and HHL sub-bands further suggests that run-length heterogeneity across different spatial-frequency components may provide complementary predictive information. In contrast, higher wavelet-LHL_firstorder_TotalEnergy values were generally associated with a lower model-predicted likelihood of recurrence. No consistent monotonic relationship was observed between feature values and SHAP values for wavelet-HHL_glrlm_GrayLevelVariance and wavelet-HHL_glszm_LargeAreaHighGrayLevelEmphasis, suggesting more complex nonlinear associations with the model output. Although these two features did not show consistent SHAP directions, the remaining four features exhibited clearer directional patterns. Taken together, greater gray-level dispersion and local texture heterogeneity within the LAC were associated with a higher model-predicted likelihood of recurrence, whereas higher TotalEnergy was generally associated with a lower predicted likelihood of recurrence. These overall patterns may be related to imaging anifestations associated with AF-related atrial remodeling.

Previous pathological and mechanistic studies have shown that AF-related atrial remodeling is commonly characterized by fibrosis, increased extracellular matrix deposition, and spatially heterogeneous alterations in atrial wall architecture (44, 45). Taken together, these studies suggest that AF-related structural and functional remodeling may further affect blood flow and the spatial distribution of contrast material within the LAC, thereby giving rise to gray-level and texture differences on CT images that may be detectable by radiomic analysis (19). All radiomic features ultimately retained in this study were derived from wavelet-transformed images rather than from the original images, suggesting that predictive information associated with AF recurrence may be at least partly encoded in subtle signal differences that can be quantified after multiscale transformation but may not be readily discernible on routine visual assessment.

For example, a higher wavelet-LHL_firstorder_TotalEnergy value indicates a greater aggregate magnitude of gray-level intensities within the ROI and may reflect an imaging phenotype characterized by relatively greater contrast opacification and a more homogeneous gray-level distribution within the LAC. In light of previous CT studies, such signal alterations may be associated with complex atrial remodeling involving AF-related inflammation, fibrosis, and fatty infiltration (46, 47). However, this feature may also be influenced by ROI extent, image sampling, and reconstruction parameters and therefore should not yet be attributed to any single, well-defined pathological process (48). Collectively, the radiomic features identified in this study may indirectly reflect, at least to some extent, the signal heterogeneity associated with AF-related atrial remodeling. Similarly, Hu et al. (24) reported that radiomic features characterizing signal-intensity distribution within LA-EAT were associated with AF recurrence after ablation. Although that study focused on LA-EAT rather than the intracavitary blood pool, both studies suggest that heterogeneity in local signal distribution may contain imaging information associated with AF recurrence after ablation. This consistent pattern across different ROIs suggests that imaging signal heterogeneity may reflect broader phenotypic manifestations of AF-related remodeling rather than changes confined to a specific tissue.

If prospectively validated, these radiomic phenotypes may facilitate the identification of subgroups at higher risk of recurrence using routine preprocedural CCTA, thereby informing individualized post-ablation surveillance and follow-up strategies, such as more frequent rhythm monitoring in high-risk subgroups, and supporting better-informed shared decision-making between clinicians and patients.

Among the clinical variables, multivariable logistic regression identified LVEF and sex as independent predictors of recurrence after AF ablation. Multiple studies have shown that reduced LVEF is associated with a higher risk of recurrence after AF ablation and may independently predict this outcome (8, 49, 50). A potential mechanism is that reduced LVEF increases left ventricular filling pressure, thereby promoting neurohumoral remodeling through activation of the renin-angiotensin-aldosterone system (RAAS). This pressure overload may be transmitted retrogradely to the LA through ventriculoatrial mechanical coupling, thereby promoting atrial fibrosis and electrical remodeling, which are key substrates for recurrence after AF ablation (51–53). Thus, in the present study, LVEF may reflect not only left ventricular systolic function but also, to some extent, the atrial loading and remodeling milieu associated with AF recurrence.

Of note, several established predictors of recurrence after AF ablation in our cohort, including AF type and LAD, were associated with recurrence in univariable analysis but were no longer independently significant in the multivariable model. This may be explained by the substantial pathophysiological overlap among these variables. Persistent AF is typically accompanied by more advanced atrial remodeling, often manifested as LA enlargement (54). As AF persists, atrioventricular mechanical dyssynchrony and a rapid, irregular, and sustained ventricular response may further aggravate hemodynamic disturbance, induce AF-related cardiomyopathy in some patients, and consequently reduce LVEF (53, 55). Because these factors are closely interrelated pathophysiologically and often highly collinear statistically, their individual effects may be attenuated when they are entered simultaneously into a multivariable model. LVEF may therefore serve as an integrative marker capturing both global cardiac function and the extent of atrial remodeling in a multivariable setting. Qiao et al. (50) reported a similar finding in an AF ablation cohort with concomitant heart failure: although non-paroxysmal AF and LAD were associated with adverse outcomes in univariable analysis, only LVEF <50% remained an independent predictor after adjustment for confounders, consistent with our findings.

Furthermore, multivariable logistic regression identified sex as an independent predictor of AF recurrence after ablation. With female sex as the reference category, male sex was associated with lower odds of recurrence (adjusted OR = 0.290, 95% CI: 0.144–0.582, *P* = 0.0005), indicating higher odds of post-ablation AF recurrence in female than in male patients. This finding is consistent with those of a previous meta-analysis of propensity score-matched studies and PVI cohort studies (56, 57). Clinically, female patients with AF often have a greater symptom burden but are less likely to undergo ablation and tend to be referred later. At the time of ablation, they are often older and have more advanced left atrial remodeling, all of which may contribute to the higher risk of post-ablation AF recurrence (58–61). Given the limited number of recurrence events in the training cohort, only the two independent predictors identified in the multivariable analysis were retained in the final clinical model to reduce the risk of overfitting and preserve model stability. This parsimonious clinical model reduces the number of required variables and may facilitate implementation in routine clinical practice.

Consistent with previous radiomics studies (62), incorporating radiomic features further improved the predictive performance of the clinical-only model in the present study. In the external validation cohort, the combined model demonstrated superior discriminative performance compared with both the clinical-only model (AUC: 0.869 vs. 0.704) and the radiomics-only model, suggesting that integration of radiomic and clinical information may improve the accuracy of preprocedural risk stratification for AF recurrence after ablation. Previous studies have also shown that combining clinical variables with ultrasound- or CCTA-derived radiomic features of the LA or LAA may improve the prediction of AF recurrence after ablation. For example, Zheng et al. (62) reported that incorporating ultrasound-based LA radiomic features into a clinical nomogram improved predictive performance, whereas Yin et al. (63) showed that a combined model integrating CCTA-derived LAA radiomic features demonstrated good predictive performance, further supporting the value of multidimensional data integration for preprocedural risk stratification for post-ablation AF recurrence. Notably, baseline characteristics and recurrence rates differed across the three cohorts (training cohort: 32.5%; internal validation cohort: 31.9%; external validation cohort: 20.6%). Such between-cohort variation is common in multicenter retrospective studies and may reflect differences in patient case mix, comorbidity burden, and clinical management strategies across centers, while also providing an opportunity to assess model robustness in diverse clinical settings. Despite these differences, the combined model maintained good discriminative performance in the external validation cohort, suggesting some degree of generalizability across centers and populations with differing event rates.

This study has several limitations. First, the overall sample size was limited, and the study was retrospective. Late AF recurrence was assessed using intermittent ECG monitoring, which may have resulted in underdetection of asymptomatic episodes. Therefore, the findings require validation in larger, prospective, multicenter cohorts with more comprehensive rhythm monitoring.

Second, CCTA-related variability may have influenced radiomic feature robustness. Despite standardized preprocessing, differences in CT scanners and acquisition protocols between the two centers may have affected the intercenter reproducibility of certain features. Irregular rhythm in patients with persistent AF may also have introduced motion artifacts and reduced cardiac-phase consistency. In addition, interpatient differences in contrast medium dose, circulatory status, and acquisition timing could not be fully standardized. Because neither correction based on blood-pool attenuation values nor enhancement-stratified sensitivity analysis was performed, residual confounding related to contrast enhancement cannot be excluded.

Third, detailed intraprocedural mapping data and quantitative ablation parameters were not available for integration. Because the ablation strategy and extent of additional ablation were individualized, treatment-level heterogeneity should be considered when interpreting recurrence outcomes.

Finally, LGE-CMR and 3D electroanatomic voltage mapping were not systematically performed, precluding direct validation of the relationship between radiomic features and atrial fibrosis or low-voltage substrate. Future prospective multimodal studies are warranted to further validate these findings and clarify the biological basis of the identified radiomic features.c mapping are warranted to clarify the biological basis of the identified radiomic features.

## Conclusion

In conclusion, based on the concept of the LAC, we developed and validated a combined CCTA radiomics-clinical model for predicting LR after RFCA in patients with AF. The model showed good discrimination and generalizability in both the internal validation cohort and the independent external validation cohort. The derived nomogram may facilitate individualized risk estimation and may serve as an adjunctive tool for preprocedural risk stratification and treatment decision-making in patients with AF.

## Data Availability

The raw data supporting the conclusions of this article will be made available by the authors, without undue reservation.
