# Supplementary material for: A computed tomography-based radiomics-clinical model incorporating left atrial and proximal pulmonary vein features predicts recurrence after radiofrequency catheter ablation of atrial fibrillation: a multicenter study
Source: Front Med (Lausanne). 2026 Sep 16;13:1935193. doi: 10.3389/fmed.2026.1935193 (PMC13623759; doi:10.3389/fmed.2026.1935193)
Supplement: Supplementary file 1 [file Table1.docx]

# Supplementary Materials

**Supplementary Table S1.** CT Acquisition Protocols for the Two

Centers (page 2)

**Supplementary Methods S1.** Detailed Description of the Deep Learning Segmentation Pipeline (page 3–4)

**Supplementary Figure S1.** Spearman Correlation Matrix of Candidate Features

(page 5)

**Supplementary Methods S2.** Formula for Calculating the Rad-score (page 6)

**Supplementary Table S2.** Quantitative evaluation of automated segmentation performance (page 7)

**Supplementary Figure S2.** Pairwise DeLong comparisons of AUCs among the five ROI-based radiomic models in the external validation set (page 8)

**Supplementary Table S3.** Definitions, Directions of Association With Model-Predicted Recurrence, and Potential Clinical Interpretations of Selected Radiomic Features (page 9–11)

**Supplementary Table S1. CT Acquisition Protocols for the Two Centers**

| **Parameter** | **Center A** | **Center B** |
| --- | --- | --- |
| Scanner model | SOMATOM Force (dual-source CT) | SOMATOM Perspective (128-slice CT) |
| Vendor | Siemens Healthineers, Germany | Siemens Healthineers, Germany |
| Scan mode | ECG-gated helical acquisition | ECG-gated helical acquisition |
| Scan range | Tracheal carina to inferior margin of diaphragm | Aortic arch to cardiac apex |
| Bolus triggering | Bolus tracking, trigger threshold 100 HU | Test bolus method for scan delay determination |
| Tube voltage | 100–120 kV, adjusted by body mass index | 120 kV |
| Tube current | Automatic tube current modulation (CARE Dose4D) | Automatic tube current modulation |
| Detector collimation | 128 × 0.6 mm | 128 × 0.6 mm |
| Reconstructed slice thickness | 0.75 mm | 0.6 mm |
| Reconstruction interval | 0.5 mm | 0.3 mm |
| Gantry rotation time | 280 ms | 280 ms |
| Matrix size | 512 × 512 | 512 × 512 |
| Reconstruction kernel | B26f (medium-smooth) | B30f |
| Contrast agent | Iohexol, 350 mgI/mL | Ioversol, 320 mgI/mL |
| Contrast volume | 50–80 mL, adjusted by body weight | 1 mL/kg (maximum 80 mL) |
| Injection rate | 4–5 mL/s | 5 mL/s |
| Saline flush | 40 mL | 20 mL |

*Despite minor inter-center differences in acquisition and reconstruction parameters, all scans provided complete coverage of the left atrial complex.

**Supplementary Methods S1. Detailed Description of the Deep Learning Segmentation Pipeline**

**S2.1 Deep Learning Segmentation Framework and Model Selection**

TotalSegmentator is a large-scale, fully automated CT anatomical segmentation tool developed by Wasserthal et al. It is built on the nnU-Net v2 framework and covers 104 classes of human anatomical structures. Its training data were derived from multiple publicly available CT datasets and routine clinical scans, all of which were manually annotated by domain experts and subjected to rigorous quality control, thereby establishing a high-quality segmentation benchmark. The tool has been validated in multiple independent studies and is currently recognized as one of the most widely used general-purpose CT segmentation tools.

Based on these advantages, we used pretrained TotalSegmentator models for target structure extraction. Given the substantial differences in boundary characteristics among cardiac substructures, a single model is insufficient to simultaneously achieve fine delineation of the cardiac chambers and preserve vascular integrity. Therefore, two distinct pretrained subtask models were used to segment different target structures:

(1) Cardiovascular subtask model (Dataset 293: TotalSegmentator_part3_cardiac): optimized for cardiovascular substructures and used to generate masks for the left atrial appendage (LAA) and pulmonary veins (PVs).

(2) High-resolution cardiac chamber subtask model (Dataset 301: heart_highres): optimized for fine boundary delineation of the cardiac chambers and used to generate the mask for the left atrial body (LA body).

**S2.2 Definition of the Left Atrial Complex (LAC)**

The LAC was defined as comprising the following three components:

(1) LA body: defined as the main smooth-walled chamber of the left atrium, corresponding to the left atrium label generated by the high-resolution cardiac chamber subtask model (Dataset 301). The default boundaries generated by the model were accepted without manual modification.

(2) LAA: defined as a finger-like projection originating from the anterior wall of the left atrium, corresponding to the LAA label generated by the cardiovascular subtask model (Dataset 293). The default boundary at the LAA neck produced by the model was accepted without modification.

(3) Proximal PV trunks: the cardiovascular subtask model (Dataset 293) generated a mask covering the entire pulmonary venous tree. During post-processing, only the proximal trunk segments of the bilateral superior and inferior PVs, extending from the ostium to the first major bifurcation, were retained. Distal branches beyond the first major bifurcation were excluded because of their greater anatomical variability, smaller caliber, and lower segmentation consistency.

**S2.3 Inference Configuration**

All segmentation inference procedures were performed on a workstation equipped with an NVIDIA RTX 4090 GPU (24 GB VRAM). The input images consisted of arterial-phase contrast-enhanced CCTA DICOM series, which were processed using the standard nnU-Net v2 preprocessing pipeline, including foreground-based cropping and Z-score normalization. Inference was performed using the 3D full-resolution (3d_fullres) configuration, and the resulting segmentations were exported in NIfTI format (.nii.gz).

**S2.4 Mask Integration**

The three independent masks, including the LA body, LAA, and proximal PV trunks, generated by the two models described above, were integrated in the native voxel space using a Boolean union operation to produce the final LAC mask, which was subsequently used for radiomic feature extraction.

Because the masks of different substructures were generated by two independent pretrained models, minor voxel-level overlaps could occur in anatomical transition zones during the merging process, primarily at the interfaces between the LA body and LAA and between the LA body and PV ostia. These overlapping voxels were preserved without additional correction and were directly incorporated into the final region of interest (ROI). This strategy was intended to avoid subjective bias introduced by manual adjustment and to maximize the objectivity and reproducibility of the fully automated segmentation pipeline.

**S2.5 Segmentation Quality Control**

All automatically generated segmentation results underwent dual-reader visual review, as described in the main text, to assess their anatomical plausibility. During this review, particular attention was paid to the following categories of obvious segmentation errors:

(1) obvious discontinuities or non-anatomical extensions of the left atrial wall contour;

(2) incomplete inclusion of the PV ostia and proximal trunk segments within the segmentation;

(3) incomplete segmentation of the LAA or abnormal fusion of its boundary with adjacent structures (eg, the left ventricle);

(4) erroneous inclusion of non-target surrounding tissues, such as the aortic root or pulmonary artery, within the LAC mask.

If any of the above abnormalities were identified, the case was considered to have failed quality control and was excluded from subsequent analyses.

**Supplementary Figure S1. Clustered heatmap of pairwise Spearman correlations among the six core radiomic features selected by LASSO regression.**

* Color intensity reflects correlation magnitude; hierarchical clustering was applied to both rows and columns.

**Supplementary Methods S2. Formula for Calculating the Rad-score**

Rad-score = 46.9798 - 0.0084 × wavelet-LHL_firstorder_TotalEnergy

+ 0.0039 × wavelet-LHL_firstorder_Variance

- 0.0379 × wavelet-LHH_glrlm_GrayLevelVariance

- 0.0150 × wavelet-HHL_glrlm_GrayLevelVariance

+ 0.0478 × wavelet-HHL_glszm_LargeAreaHighGrayLevelEmphasis

+ 0.0210 × wavelet-HHL_glszm_SizeZoneNonUniformity

*In the feature names, “wavelet-XXX” indicates that the feature was extracted from the corresponding sub-band generated by three-dimensional wavelet decomposition, where L and H denote low-pass and high-pass filtering, respectively; the final model retained features from the LHL, LHH, and HHL sub-bands.

**Supplementary Table S2. Quantitative evaluation of automated segmentation performance**

A. **Dice coefficients of the segmentation models on their respective validation sets**

| **Structure** | **Model** | **Dice coefficient** |
| --- | --- | --- |
| LA body | Dataset301 | 0.9763 |
| LAA | Dataset293 | 0.9665 |
| PV trunk | Dataset293 | 0.9352 |

B. **Comparison of automated segmentation with manual reference annotations in 30 patients**

| **Structure** | **Dice** | **IoU** |
| --- | --- | --- |
| LAC | >97.4% | >95% |

*Part A reports the Dice coefficients achieved by the segmentation models on their respective validation sets to document their segmentation performance. Part B reports the overall LAC segmentation results obtained by comparing automated segmentation with manual reference annotations in 30 patients, demonstrating a high level of agreement between the two methods.

Abbreviations: LA, left atrium; LAA, left atrial appendage; PV, pulmonary vein; LAC, left atrial complex; IoU, intersection over union.

**Supplementary Figure S2. Pairwise DeLong comparisons of AUCs among the five ROI-based radiomic models in the external validation set**

*The matrix shows p values from pairwise DeLong tests comparing the AUCs of the five ROI-based radiomic models. Cell colors represent the corresponding p values, as indicated by the color scale. For comparisons involving the LAC model, its AUC was significantly higher than those of the LAA model (p = 0.024), PV trunk model (p = 0.008), and LA body + LAA model (p = 0.027), whereas the difference between the LAC and LA body models was not statistically significant (p = 0.314). AUC, area under the receiver operating characteristic curve; LAC, left atrial cavity; LA body, left atrial body; LAA, left atrial appendage; PV trunk, pulmonary vein trunk.

**Supplementary Table S3. Definitions, Directions of Association With Model-Predicted Recurrence, and Potential Clinical Interpretations of Selected Radiomic Features**

| **Feature** | **Mathematical Definition** | **Association With Model Prediction** | **Potential Clinical Interpretation** |
| --- | --- | --- | --- |
| firstorder_TotalEnergy (LHL) | Sum of the products of squared voxel values and voxel volume within the LHL sub-band, reflecting the overall signal energy in this sub-band and being influenced by both gray-level intensity and ROI volume | Lower predicted probability | May reflect the overall status of contrast filling and blood-pool signal within the LAC; higher energy may indicate relatively more complete contrast filling and more favorable intracavitary flow exchange, potentially corresponding to a more favorable left atrial functional state |
| firstorder_Variance (LHL) | Variance of voxel values around their mean within the LHL sub-band, reflecting the dispersion of the gray-level distribution | Higher predicted probability | Greater gray-level dispersion may indicate more heterogeneous intracavitary contrast filling, potentially related to local flow stasis or incomplete mixing and indirectly reflecting impaired left atrial hemodynamics. |
| glszm_SizeZoneNonUniformity (HHL) | Degree of nonuniformity in the distribution of connected zones of different sizes in the GLSZM of the HHL sub-band | Higher predicted probability | May reflect the degree of spatial "patchiness" in contrast distribution; higher values may suggest the coexistence of large homogeneous zones and scattered fragmented zones, indicating more irregular spatial organization of contrast mixing and possibly more severe hemodynamic disturbance |
| glrlm_GrayLevelVariance (LHH) | Variance in gray-level values across runs in the GLRLM within the LHH sub-band; sensitive to fine-scale gray-level edges and textural variations | Higher predicted probability | May capture subtle local variations in contrast distribution that are difficult to appreciate on conventional visual assessment; more pronounced fine-scale signal variations may suggest more complex local intracavitary flow patterns, possibly reflecting left atrial functional and structural remodeling |
| glrlm_GrayLevelVariance (HHL) | Variance in gray-level values across runs in the GLRLM within the HHL sub-band, reflecting local textural heterogeneity along a different spatial orientation | Non-monotonic association | Shares the same metric as the LHH-derived Gray Level Variance; their joint selection suggests that nonuniform contrast mixing may exhibit directional dependence; different patients may manifest textural heterogeneity along different spatial orientations, which may explain the non-monotonic association with recurrence |
| glszm_LargeAreaHighGrayLevelEmphasis (HHL) | Measure emphasizing the joint distribution of larger connected zones with higher gray-level values in the GLSZM within the HHL sub-band | Non-monotonic association | Quantifies the relative proportion of large, high-gray-level connected zones in the filtered image; its biological correlate in the blood pool remains unclear: it may reflect either adequate contrast mixing (unimpeded flow) or localized contrast pooling (flow stasis), and this bidirectional interpretability may explain the non-monotonic association with recurrence |

***** All features were extracted from wavelet-transformed images of the intracavitary ROI within the LAC on contrast-enhanced cardiac CT. L and H denote low-pass and high-pass filter components, respectively; sub-band labels (e.g., LHL) correspond to the filter type applied along the x, y, and z directions in sequence. The ROI in this study represents the contrast-enhanced blood pool rather than atrial wall tissue; therefore, the features described herein characterize the spatial distribution patterns of contrast within the LAC lumen, and wavelet coefficients do not directly correspond to original HU values.
Associations in Column 3 reflect the direction of feature contributions to model-predicted recurrence probability based on SHAP analysis and should not be interpreted as independently validated prognostic associations.The potential clinical interpretations in Column 4 are hypothesis-generating in nature and should not be regarded as direct measures of atrial hemodynamics, fibrosis, or specific pathophysiological mechanisms. The degree of contrast mixing within the blood pool is influenced by LA flow velocity, mechanical function, and chamber geometry (S1); reduced LAA emptying velocity, decreased LA strain, and increased LA volume have all been demonstrated to be independently associated with risk of AF recurrence after ablation (S2–S4); the above references serve only as a pathophysiological framework and do not constitute direct validation of the radiomic features in this study. The "zones" in the GLSZM are mathematically defined connected regions based on gray-level similarity of adjacent voxels and do not represent actual anatomic lesions or fibrotic regions.

**Abbreviations:** GLRLM = gray-level run-length matrix; GLSZM = gray-level size zone matrix; LAC = left atrial complex; ROI = region of interest; SHAP = SHapley Additive exPlanations; LA = left atrium; LAA = left atrial appendage; AF = atrial fibrillation; HU = Hounsfield unit.

**References**

S1. Guha A, Dunleavy MP, Hayes S, Afzal MR, Daoud EG, Raman SV, et al. Accuracy of contrast-enhanced computed tomography for thrombus detection prior to atrial fibrillation ablation and role of novel Left Atrial Appendage Enhancement Index in appendage flow assessment. Int J Cardiol. (2020) 318:147-52. doi:10.1016/j.ijcard.2020.06.035

S2. Thotamgari SR, Sheth AR, Ahmad J, Bawa D, Thevuthasan S, Babbili A, et al. Low left atrial appendage emptying velocity is a predictor of atrial fibrillation recurrence after catheter ablation. J Cardiovasc Electrophysiol. (2022) 33(8):1705-11. doi:10.1111/jce.15580

S3. Nielsen AB, Skaarup KG, Djernæs K, Hauser R, San José Estépar R, Sørensen SK, et al. Left atrial contractile strain predicts recurrence of atrial tachyarrhythmia after catheter ablation. Int J Cardiol. (2022) 358:51-7. doi:10.1016/j.ijcard.2022.04.056

S4. Bajraktari G, Bytyçi I, Henein MY. Left atrial structure and function predictors of recurrent fibrillation after catheter ablation: a systematic review and meta-analysis. Clin Physiol Funct Imaging. (2020) 40(1):1-13. doi:10.1111/cpf.12595
